# Multiparametric magnetic resonance imaging-based comprehensive model on prediction of lymphovascular space invasion in cervical cancer

**DOI:** 10.3389/fonc.2025.1578119

**Published:** 2025-10-06

**Authors:** Tao Yang, Qinqin Yi, Jingshan Gong

**Affiliations:** ^1^ The Second Clinical Medical College of Jinan University, Department of Radiology, Shenzhen People’s Hospital, Shenzhen, Guangdong, China; ^2^ Department of Radiology, Shenzhen People’s Hospital, The First Affiliated Hospital of Southern University of Science and Technology, The Second Clinical Medical College of Jinan University, Shenzhen, Guangdong, China

**Keywords:** cervical cancer, lymph vascular space invasion, radiomics, deep learning, magnetic resonance imaging, machine learning

## Abstract

**Objective:**

To develop and validate a comprehensive model integrating multiparametric magnetic resonance imaging (MRI) radiomics and deep learning features for preoperative prediction of LVSI in early-stage cervical cancer.

**Methods:**

155 patients from January 2019 to December 2023 were enrolled in this study and divided into the training and validation cohorts randomly at a ratio of 7:3. Radiomics and deep learning features were extracted from T2-weighted images (T2WI), apparent diffusion coefficient (ADC) maps, and late contrast-enhanced T1-weighted images (CE-T1WI). Mann–Whitney U test, the least absolute shrinkage and selection operator regression (LASSO) were used to select radiomics and deep learning features. Radiomics model (Rad model), deep learning model (DL model), and radiomics-deep learning model (RDL model) were derived from the training cohort using support vector machines (SVM) classifier. The prediction performances of the three models were evaluated with the area under the curve (AUC), calibration curve, decision curve analysis (DCA) and tested in the validation cohort.

**Results:**

The RDL model achieved predictive performance for LVSI in cervical cancer with an AUC of 0.968 (95% confidence interval (CI): 0.938-0.999) in the training cohort, higher than 0.801(95% CI: 0.712-0.891) of Rad model and 0.902(95 CI: 0.845-0.959) of DL model with statistical significance after Bonferroni correction. In the validation cohort, the predictive performance of the fusion model (RDL)(AUC = 0.859, 95% CI 0.751-0.967) was significantly superior to that of the single model (AUC of DL Model = 0.745 95% CI 0.595-0.894; AUC of Rad Model = 0.686 95% CI 0.525-0.847, P < 0.001), however, the DL and radiomics models did not demonstrate statistically significant differences in performance within the validation cohort (Delong test, P>0.05). Analysis of the calibration and decision curves indicated superior predictive precision and net clinical benefit for the RDL model relative to the others.

**Conclusions:**

The advanced RDL model demonstrated strong predictive accuracy for LVSI in cervical cancer, suggesting its promising role as a noninvasive imaging biomarker. This tool could significantly enhance preoperative treatment planning by providing reliable insights without invasive procedures.

## 1 Introduction

Cervical cancer remains one of the most significant cancer threats to women’s health globally, maintaining its position as the gynecological malignancy with both the highest incidence and mortality rates worldwide. According to 2022 global statistics, it is estimated that there were 111,820 new diagnoses and 61,579 deaths in China and 13,740 new diagnoses and 5,830 deaths in the United States in 2022 (1, 2). Furthermore, the incidence and mortality rates of cervical cancer in China have witnessed a significant increase since 2000 (2). Lymph-vascular space invasion (LVSI) is defined as the dissemination of neoplastic cells within lymphatic and/or blood vessels (3, 4), and has been reported to be strongly correlated with lymph node metastasis and poor prognosis (5–9). While lymphovascular space invasion (LVSI) doesn’t substantially influence the clinical staging of cervical cancer, treatment strategies diverge significantly between LVSI-positive and LVSI-negative patients, as outlined by the National Comprehensive Cancer Network (NCCN) protocols. Surgeons often tailor their approach based on this histological feature, even though it doesn’t alter the disease’s formal classification. Specifically, patients without LVSI have the option to choose a fertility-sparing treatment strategy, thereby avoiding the need for radical hysterectomy (10, 11). Therefore, preoperative acknowledge of LVSI status is significant for treatment plan decision. However, accurate identification of LVSI can only be achieved through a detailed pathological examination conducted after hysterectomy.

Magnetic resonance imaging, with high soft-tissue resolution, is an important component in the diagnosis and staging of cervical cancer, but it still cannot provide intuitive information to identify LVSI status. As a result, an increasing number of researchers are employing MRI-based radiomics models to predict LVSI status in CC, and a large number of original studies have been published, many results are encouraging (12–18). However, the low-order nature of radiomics features may limit their ability to characterize the heterogeneity of medical images. Recently, deep learning has become an emerging field on medical image processing problems. Convolutional Neural Networks, or CNNs, are particularly adept at identifying more advanced features in medical images while preserving essential spatial data–crucial factor for enhancing medical diagnosis when stacked up against radiomics approaches (19, 20). However, the main current constraint on the performance of deep learning models is limited training data. A previous study indicated that the combination of radiomics and deep learning features has great potential on using limited data to predict LVSI status in CC (21), yet the results were not satisfactory.

Lymphovascular space invasion (LVSI) serves as a key prognostic marker for cervical cancer progression. Yet, the diagnostic potential of deep learning-derived features in assessing LVSI remains underexplored. This study aims to bridge that gap by systematically comparing radiomic and deep learning feature extraction techniques. We developed and validated an integrated predictive model leveraging multiparametric MRI data to noninvasively determine LVSI status in early-stage cervical cancer patients, incorporating cross-modal imaging features for enhanced diagnostic accuracy.

## 2 Materials and methods

### 2.1 Patient population

**Figure 1:**
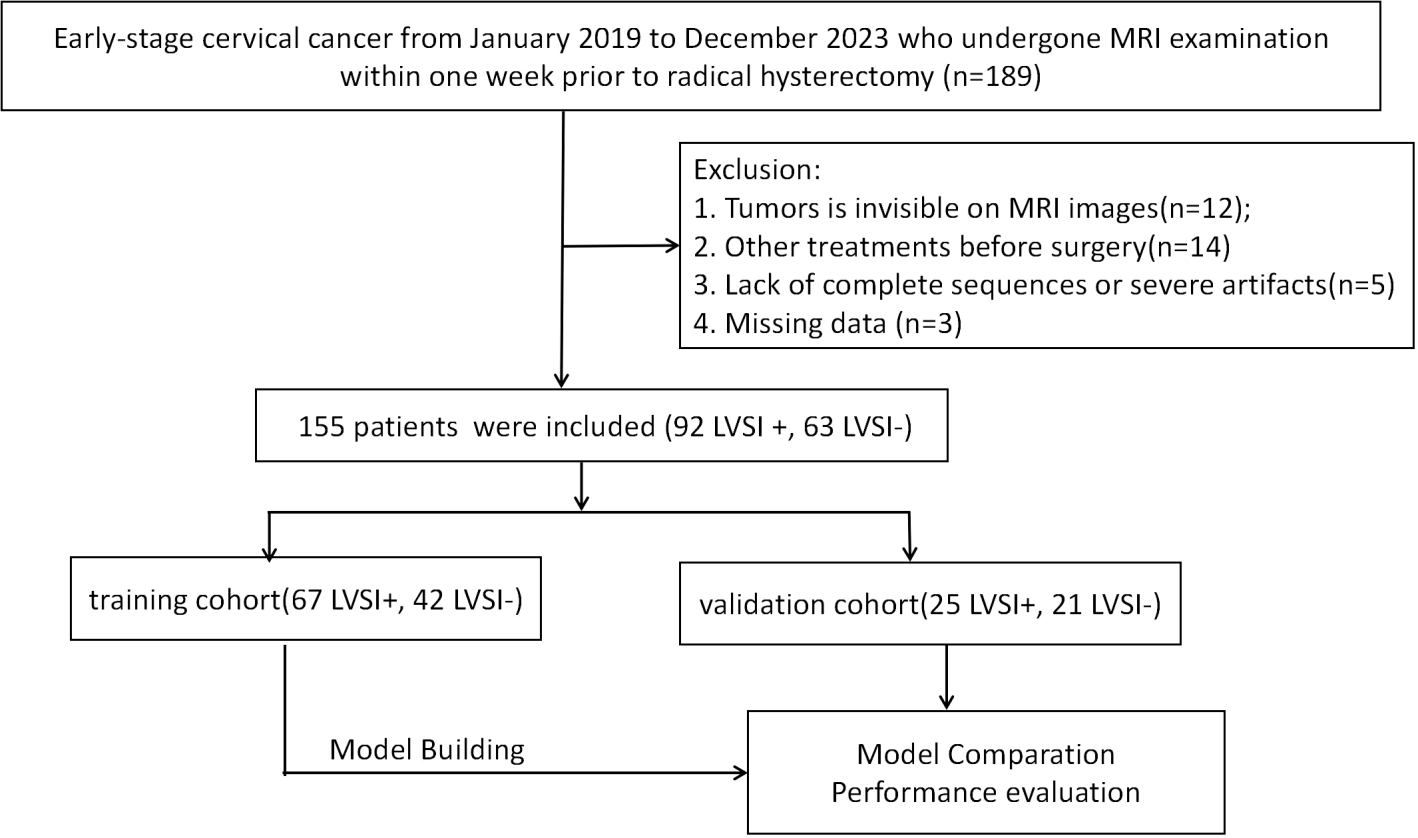
The flowchart of patient selection.

This retrospective study received approval from the institutional ethics review board, which waived the need for patient consent or written authorization. The research cohort comprised 155 individuals diagnosed with cervical cancer through pathological confirmation, with cases drawn from January 2019 through December 2023. The inclusion criteria of this study were as follows: (1) patients who underwent a pelvic MRI examination within one week before operation, (2) no history of preoperative treatment, (3) diagnosed by postoperative pathology with complete clinical data. Exclusion criteria were as follows: (1) images with severe motion artifacts or evident noise; (2) tumors were invisible, (3) combination with other tumor diseases. Participants were assigned to either training or validation cohorts (7:3 stratified ratio, random assignment). Process depicted in **Figure 1**.

We collected these patients’ images and clinical data from our hospital’s regular clinical records and picture archiving and communication systems (PACS). We retrospectively analyzed their clinical information including age, maximum tumor diameter, menopausal status, HPV infection status, reproductive history and various serum markers including squamous cell carcinoma antigen (SCC-Ag).

### 2.2 MRI protocol

The MRI scans were conducted using either a 3.0T or 1.5T scanner in the supine posture with an 18-channel abdominal-phased array coil. For contrast-enhanced imaging, gadolinium diethylenetriamine penta-acetic acid was administered intravenously at a standard dose of 0.1 mmol/kg, injected at a rate of 3 milliliters per second. The imaging protocol included axial T2WI, axial diffusion-weighted imaging (DWI), sagittal contrast enhanced T1WI. The parameters are as follows: (1) Axial T2-weighred images: repetition time(TR), 4000ms; echo time (TE), 109ms; slice thickness, 5mm; fields of view (FOV), 549×250mm; pixels size, 0.5×0.5 mm; spacing between slices, 6.75mm; acquisition matrix, 256×256. (2) Axial diffusion-weighted images:TR, 3000ms; TE, 84ms; slice thickness, 5mm; FOV, 549×250mm; pixels size, 2.34×2.34mm; spacing between slices, 6.75mm; acquisition matrix, 128×104. (3) Sagittal contrast-enhanced T1- weighted images: TR, 5.77ms; TE, 2.66ms; slice thickness, 2.5mm; FOV, 703×320mm; pixels spacing, 1.25×1.25mm; spacing between slices, 2.5mm; acquisition matrix, 256×146.

### 2.3 Image preprocessing and segmentation

All images were stored in Digital Imaging and Communications in Medicine (DICOM) format. Prior to analysis, all images underwent a process of resampling and standardization to ensure the reliability and repeatability of the results finally obtained. Without access to patients’ histopathological data, two radiologist-each boasting a minimum of five years’ experience in medical imaging diagnosis -independently performed manual segmentation of the regions of interest. They utilized ITK-SNAP software (Version 3.8.0, available at http://www.itksnap.org) to meticulously outline the target areas slice by slice. Both specialists remained unaware of pathological findings throughout the segmentation process to maintain objectivity. A radiologist with over 15 years of experience in radiological diagnosis validated the manual delineations. The delineations of ROIs were stored in the NIfTI (Neuroimaging Informatics Technology Initiative) format as a mask for subsequent analysis. thirty patients were randomly selected for ROI resegmentation by the radiologists 1 after one month to investigate the stability and reproducibility of extracted features.

### 2.4 Radiomics handcrafted feature extraction

Using the pyradiomics toolkit, we generated a total of 4,227 manually engineered radiomic features across three imaging modalities for each tumor region. These features fall into three distinct categories: (1) geometric, (2) intensity-based, and (3) textural characteristics. Geometric features capture the tumor’s three-dimensional morphological properties, while intensity features quantify the first-order statistical patterns of voxel values within the lesion. Textural features, derived through multiple computational approaches, reveal higher-order spatial relationships between intensity values. Specifically, we employed four established texture analysis techniques: gray-level co-occurrence matrix (GLCM), gray-level run length matrix (GLRLM), gray-level size zone matrix (GLSZM), and neighborhood gray-tone difference matrix (NGTDM) methodologies.

### 2.5 Deep learning-based feature extraction

We implemented ResNet-50 network which consist of 49 convolutional layers and one fully connected layer with 2048 neurons as the core convolutional neural network architecture to extract deep learning-based features. To optimize training efficiency despite limited data availability, transfer learning strategy was employed. Model weights were initialized using ImageNet pretraining. This study utilized the maximum ROI of the whole tumor originated from T2WI, DWI and CE-T1WI as inputs for CNN model training. Real-time augmentation techniques (random horizontal flipping/cropping) were incorporated during training. A total of 6144 deep learning-based features were finally obtained for further analysis. To improve model interpretability, Gradient-weighted Class Activation Mapping (Grad-CAM) was implemented for visual analysis. These class activation mappings were generated by leveraging gradient data derived from the CNN’s terminal convolutional layer which highlight the most important regions associated with the LVSI status in the images during the deep learning decision-making process.

### 2.6 Feature selection

**Figure 2:**
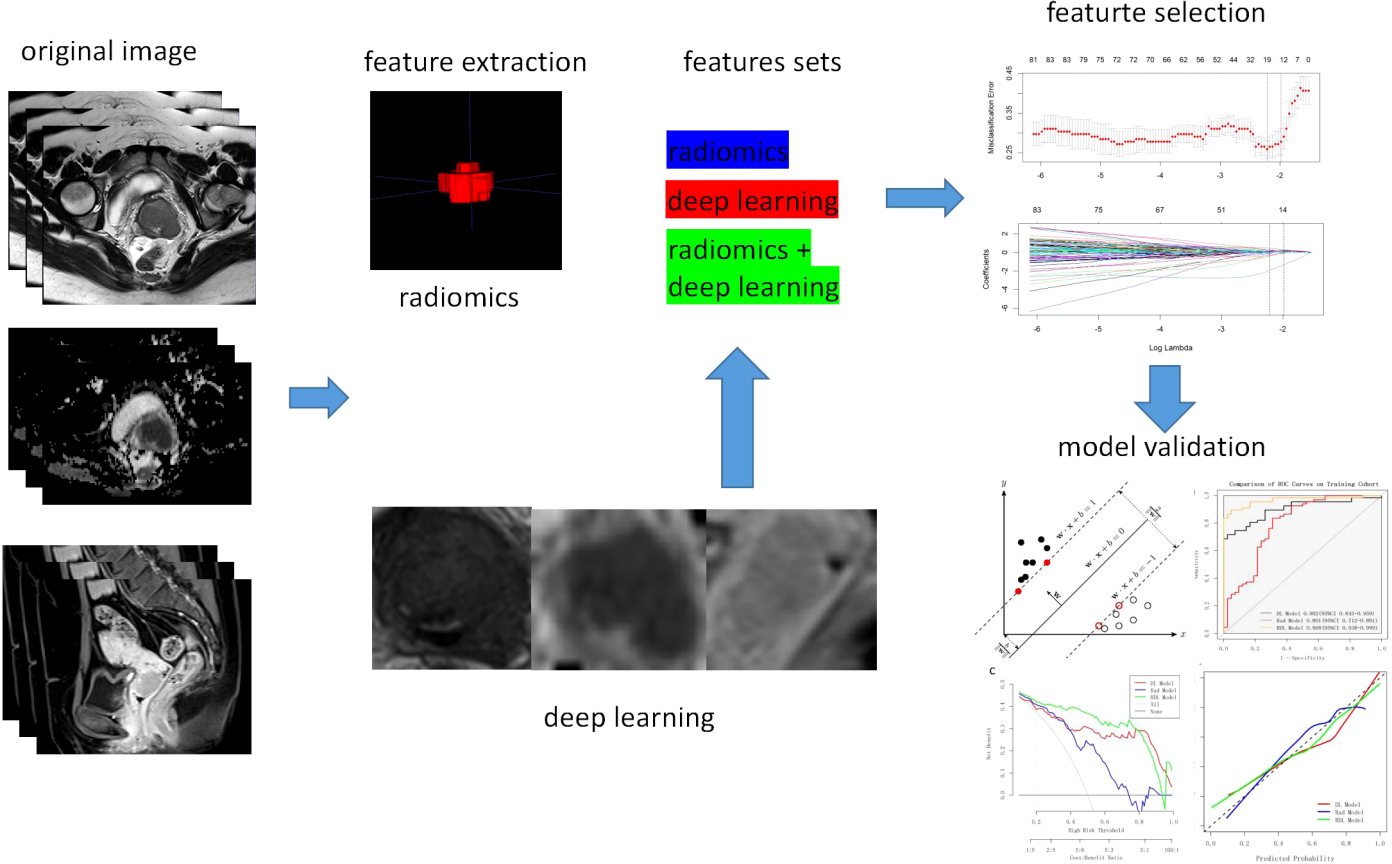
The flowchart of model construction and assessment.

Three sets of fusion features were generated based on the fusion strategy at the feature level across modalities (T2WI, ADC and CE-T1WI) or across feature types (traditional radiomics methods and deep learning methods). Feature selection process was as follows: first, features with Intraclass correlation coefficient(ICC) <0.75 and zero variance were screened, and all continuous features were subjected to Z-score standardization, scaled to a range of 0-1. Afterwards, Mann–Whitney U test was performed to select statistically significant features (P < 0.05). At last, the least absolute shrinkage and selection operator (LASSO) regression was performed utilizing 10-fold cross-validation to select the λ value, adhering to the one standard error (1SE) criterion to eliminate redundant and irrelevant features. The same feature selection process was applied to the three independent feature sets to select the most predictive features. The detailed procedure is presented in **Figure 2**.

### 2.7 Development of the radiomics models

Following feature selection, a Support Vector Machine (SVM) prediction model was constructed. Hyperparameter optimization was performed via grid search, focusing on the penalty parameter C (explored over the logarithmic range 10^−^³ to 10³). The kernel function types evaluated was radial basis function (RBF). ROC curve analysis assessed model performance, with the Youden Index determining the optimal cutoff. The area under the curve (AUC), accuracy, sensitivity, specificity, positive prediction value (PPV), and negative prediction value (NPV) were also calculated to further evaluate the performance of the model. DeLong’s test was employed to assess statistically significant differences between support vector machine (SVM) prediction models constructed using distinct feature types. The calibration curves were plotted using the Hosmer–Lemeshow (H–L) test, which measured how close the prediction outcome generated by the predictive model was to the observation value. Decision curve analysis (DCA) was also employed to quantify the net benefits associated with different threshold probabilities to evaluate the model’s clinical efficiency. Feature selection and model construction were performed on the training set and then validated on the test set.

### 2.8 Statistical analysis

For this study, all statistical analyses were performed using R (v4.2.3). Continuous variables, reported as mean ± standard deviation, were analyzed using either Student’s t-test or the Mann-Whitney U test, depending on data distribution. Categorical variables, expressed as proportions, were evaluated with either chi-square or Fisher’s exact tests. A 95% confidence interval was used throughout, with statistical significance set at p < 0.05 for all two-tailed tests.

## 3 Results

### 3.1 Clinical characteristics

**Table 1** outlines the clinical profiles of the patients studied. While most traits showed no notable variation between the LVSI-positive and LVSI-negative groups, tumor size and invasion depth stood out as exceptions in the training cohort. The validation cohort, however, revealed statistically significant disparities in invasion depth, lymph node involvement, and SCC levels. Across both the training and validation cohorts, all other characteristics remained comparable between patients with and without LVSI.

**Table 1:** Characteristics of cervical cancer patients in training and validation cohorts.

Characteristics	Training cohort (N = 109)			Validation cohort (N = 46)		
	LVSI- (N = 42)	LVSI+ (N = 67)	P	LVSI- (N = 21)	LVSI+ (N = 25)	P
Maximum tumor diameter (mm), mean ± SD	25.79 ± 10.50	36.10 ± 13.74	<.001	25.43 ± 23.22	32.92 ± 10.33	.153
Age (years), mean ± SD	51.10 ± 11.7	52.88 ± 10.69	.415	49.24 ± 9.74	51.36 ± 9.05	.448
PLR, n, mean ± SD	151.16 ± 76.39	183.00 ± 93.07	.066	140.85 ± 44.89	154.23 ± 38.51	.282
NLR, n, mean ± SD	3.09 ± 2.62	3.24 ± 3.31	.807	2.35 ± 1.43	3.09 ± 3.49	.364
Gestation, n, mean ± SD	3.74 ± 1.56	3.97 ± 1.69	.474	3.24 ± 1.70	3.80 ± 1.44	.232
Parturition, n, mean ± SD	2.40 ± 1.33	2.64 ± 1.55	.415	1.76 ± 1.26	2.28 ± 1.02	.131
Abortion, n, mean ± SD	1.31 ± 1.24	1.31 ± 1.29	.988	1.24 ± 1.18	1.32 ± 1.44	.836
FIGO Stage			.173			.150
stage I	24 (57.1%)	26 (38.8%)		14 (66.7%)	15 (60%)	
stage II	13 (31%)	29 (43.3%)		7 (33.3%)	6 (24%)	
stage III	5 (11.9%)	12 (17.9%)		0 (0%)	4 (16%)	
Histological type			.264			1.000
Squamous cell carcinoma	35 (83.3%)	57 (85.1%)		12 (57.1%)	22 (88%)	
Adenocarcinoma	7 (16.7%)	7 (10.4%)		9 (42.9%)	3 (12%)	
Adenosquamous carcinoma	0 (0%)	3 (4.5%)		0 (0%)	0 (0%)	
Degree of cellular differentiation			.361			1.000
High	2 (4.8%)	1 (1.5%)		0 (0%)	0 (0%)	
Middle	32 (76.2%)	47 (70.1%)		18 (85.7%)	21 (84%)	
Low	8 (19%)	19 (28.4%)		3 (14.3%)	4 (16%)	
Depth of invasion			<.001			.022
< 1/3	20 (47.6%)	8 (11.9%)		12 (57.1%)	5 (20%)	
> 1/3	22 (52.4%)	59 (88.1%)		9 (42.9%)	20 (80%)	
Parametrial Involvement			1.000			1.000
No	42 (100%)	66 (98.5%)		21 (100%)	25 (100%)	
Yes	0 (0%)	1 (1.5%)		0 (0%)	0 (0%)	
Lymph node involvement			.266			.049
No	38 (90.5%)	54 (80.6%)		21 (100%)	19 (76%)	
Yes	4 (9.5%)	13 (19.4%)		0 (0%)	6 (24%)	
SCC			.535			.003
Not Elevated	19 (45.2%)	25 (37.3%)		12 (57.1%)	3 (12%)	
Elevated	23 (54.8%)	42 (62.7%)		9 (42.9%)	22 (88%)	
Menopausal_status			.690			.587
Menstruation	20 (47.6%)	28 (41.8%)		11 (52.4%)	10 (40%)	
Menopause	22 (52.4%)	39 (58.2%)		10 (47.6%)	15 (60%)	
HPV Infection			.229			1.000
No	11 (26.2%)	10 (14.9%)		5 (23.8%)	6 (24%)	
Yes	31 (73.8%)	57 (85.1%)		16 (76.2%)	19 (76%)	

LVSI, lymph-vascular space invasion; SD, standard deviation; FIGO, Federation International of Gynecology and Obstetrics; SCC, squamous cell carcinoma antigen; HPV, Human Papillomavirus. PLR, Platelet-to-Lymphocyte Ratio; NLR, Neutrophil-to-Lymphocyte Ratio.

### 3.2 Feature extraction and selection

**Figure 3:**
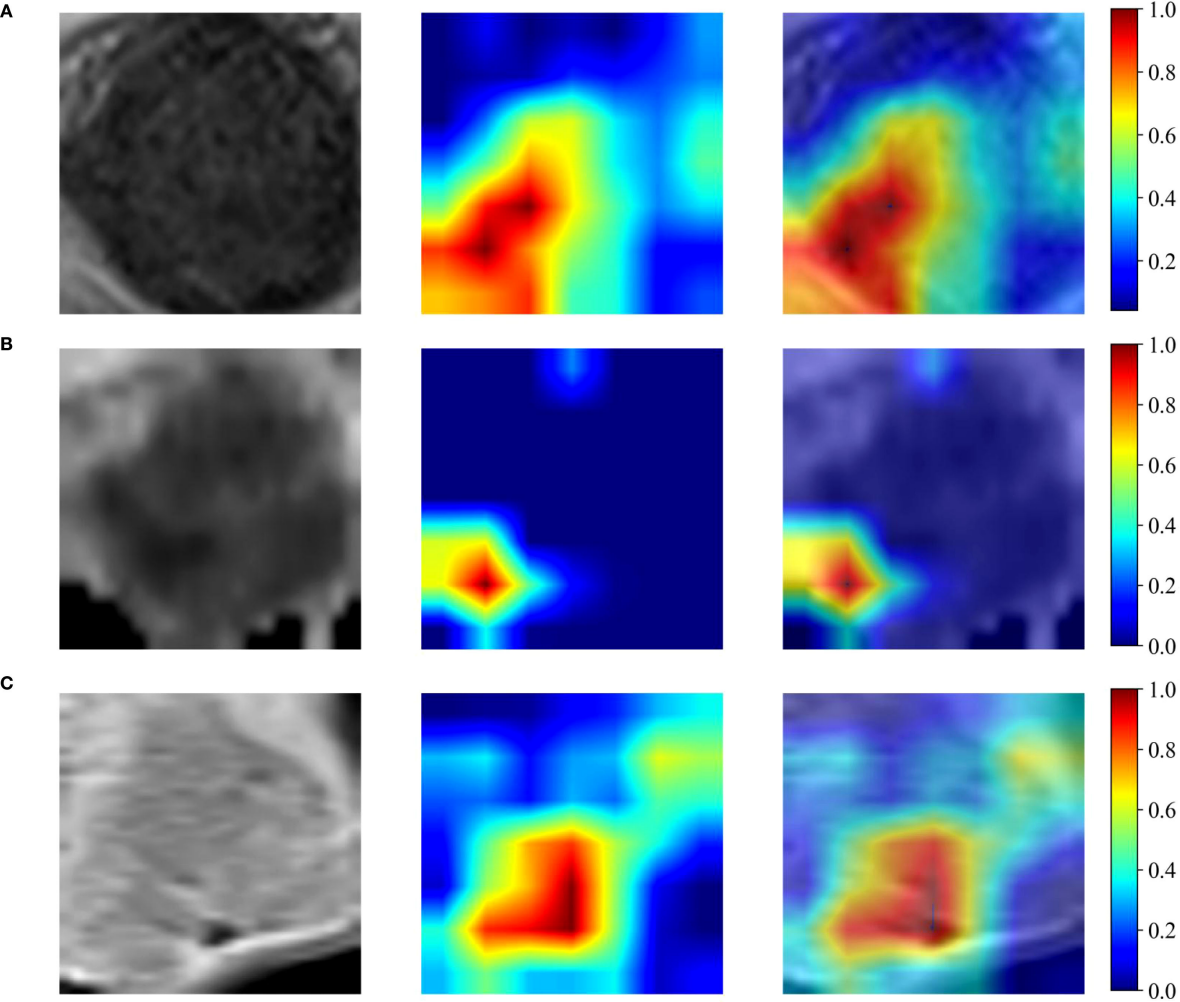
Grad-CAM heatmap. **(A-C)** shows the input image and its corresponding Grad-CAM heatmap for the T2WI, ADC maps and CE-T1WI respectively for the same patient.

For each patient, a total of 4227 hand-crafted features and 6144 deep learning-based features were extracted from the maximum ROIs of T2WI, ADC and CE-T1WI. **Figure 3A-C** shows the input image and its corresponding Grad-CAM heatmap from the same patient, highlighting the areas of interest which the deep learning model pays most attention to when extracting deep learning features related to LVSI. The Grad-CAM map for T2WI demonstrates the deep learning model focused on regions of cervical stromal ring disruption which indicated structural compromise, it potentially associated with tumor invasion patterns. For ADC, salient activation localizes to diffusion-restricted zones along vascular courses at the tumor periphery which may highlight areas correlating with tumor thrombus location. For CE-T1WI, the map prioritizes spiculated enhancement at the tumor margin which may reflect heightened neovascular activity. Notably, the ADC sequence contributed highest weight to LVSI prediction, consistent with the pathological mechanism of diffusion restriction secondary to tumor thrombi. **Table 2** delineates the range of features chosen (from 4 to 14) across various feature sets, which are subsequently employed in the SVM models’ training process.

**Table 2:** Prediction performance of SVM-based models.

Model Name	Feature size	Cohort	AUC (95%CI)	ACC	SEN	SPE	NPV	PPV
RDL Model	14	training cohort	0.968(0.938-0.999)	0.908	0.866	0.976	0.820	0.983
		test cohort	0.859(0.751-0.967)	0.826	0.760	0.905	0.760	0.905
DL Model	5	training cohort	0.902(0.845-0.959)	0.817	0.716	0.976	0.683	0.980
		test cohort	0.745(0.595-0.894)	0.717	0.840	0.571	0.750	0.700
Rad Model	4	training cohort	0.801(0.712-0.891)	0.771	0.821	0.691	0.707	0.809
		test cohort	0.686(0.525-0.847)	0.739	0.960	0.476	0.686	0.909

AUC, area under receiver operating characteristic curve; CI, confidence interval; ACC, accuracy; SEN, sensitivity; SPE, specificity; PPV, positive predictive value; NPV, negative predictive value; Rad, radiomics; DL, deep learning; RDL, radiomics and deep learning.

### 3.3 Performance comparison of model

**Table 2** presents the receiver operating characteristic (ROC) analysis outcomes across the three models. For LVSI prediction, the Rad model demonstrated AUCs of 0.801 (95%CI: 0.712-0.891) in training cohort and 0.686 (95%CI: 0.525-0.847) in validation cohort. Meanwhile, the DL model yielded stronger performance, with AUCs of 0.902 (95%CI: 0.845-0.959) and 0.745 (95%CI: 0.595-0.894) in the training and validation cohorts, respectively. The RDL model outperformed both, achieving exceptional discrimination with an AUC of 0.968 (95% CI: 0.938-0.999) in training cohort and 0.859 (95% CI: 0.751-0.967) in validation cohort.

**Figure 4:**
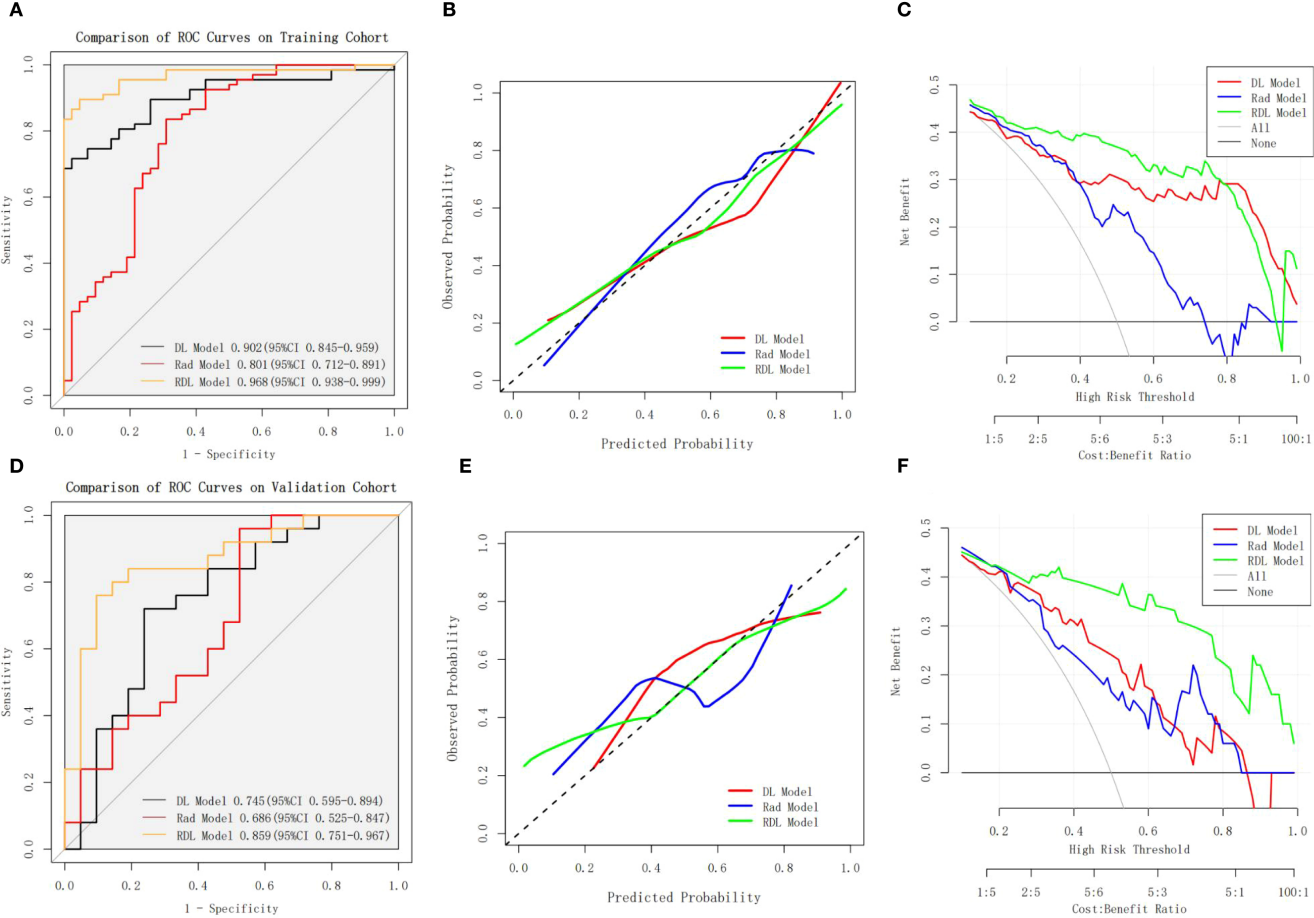
Performance Comparison of the Rad Model, DL Model, and RDL Model. **(A, B)** shows the ROC curves, calibration curves and DCA curves of the Rad Model, DL Model, and RDL Model on the training cohort; **(D-F)** shows the ROC curves, calibration curves and DCA curves of the Rad Model, DL Model, and RDL Model on the validation cohort.

Statistical comparisons revealed the RDL model’s superiority over both the Rad and DL models in training cohort (Bonferroni-adjusted p=0.000158 and p = 0.001093, respectively), though this advantage did not hold in the validation cohort (p > 0.05). ROC curves for all models in both cohorts are illustrated in **Figures 4A, D**.

In terms of diagnostic metrics, the RDL model boasts accuracy of 0.908, sensitivity of 0.866, specificity of 0.976, NPV of 0.820, and PPV of 0.983 in training cohort, and corresponding values of 0.826, 0.760, 0.905, 0.760, and 0.905 in validation cohort.

The calibration curves for RDL model demonstrated good agreement between actual results and the probabilities estimated (**Figures 4B, E**), and the Hosmer-Lemeshow test showed nonsignificant statistical difference in the training cohort (P = 0.935) and validation cohort (P = 0.504). The decision curve analysis (DCA) results for the RDL model demonstrated consistent clinical value, with a positive net benefit spanning a broad spectrum of probability thresholds in both training and validation groups (**Figures 4C, F**). This robust performance underscores the model’s practical usefulness in assessing LVSI risk for patients with early-stage cervical cancer.

## 4 Discussion

This research introduces a hybrid RDL model that integrates traditional radiomics features with deep learning-derived features to predict lymph-vascular space invasion (LVSI) in cervical cancer patients. The model showed strong predictive capabilities, achieving an area under the curve (AUC) of 0.968 (95% CI, 0.938-0.999) in the training cohort and 0.859 (95% CI, 0.751-0.967) in the validation cohort. Our approach uses a three-step decision process: first, locating structural damage using T2-weighted imaging; second, identifying restricted diffusion via ADC mapping; and third, evaluating new blood vessel formation with contrast-enhanced T1-weighted imaging. This mirrors the actual pathological progression of LVSI, allowing us to predict its presence. Notably, the ADC mapping may be able to pick up on micrometastatic deposits that were too small to find with the naked eye. In short, our findings suggest that the RDL model offers a promising imaging biomarker for predicting LVSI in cervical cancer, which could ultimately lead to better treatment decisions for patients.

Both in the worldwide and China, cervical cancer is still the biggest threat to women’s health, its incidence is still rising, and the age of onset is decreasing. With the increasing childbearing age of women in modern society, the clinical demand for early fertility preservation surgery has not been fully met. Therefore, it is a long-term and arduous task to improve the rate of fertility preservation surgery for patients with early cervical cancer. Although LVSI does not affect the clinical stage of cervical cancer, it can affect the prognosis of early stage CC and have an impact on surgical strategy selection (22). According to the NCCN guidelines, surgery is the mainstay of treatment for early-stage cervical cancer, and for patients without LVSI at clinical stage IA, only cervical conization is required, which avoids radical hysterectomy and allows for fertility preservation. The presence of lymphovascular space invasion (LVSI) has been linked to a higher risk of lymph node metastasis, which unfortunately bodes poor for patient outcomes. As a result, patients diagnosed with early-stage cervical cancer who test positive for LVSI typically require lymph node dissection to determine the extent of lymph node involvement. Consequently, being able to accurately predict LVSI before surgery could be a real game-changer, paving the way for more targeted and effective treatment strategies and a more precise prediction of how the patient will ultimately fare.

Detecting lymphovascular space invasion (LVSI) through standard imaging methods remains difficult since it’s a microscopic histopathological feature. Although research has linked LVSI to tumor size, parametrial involvement, stromal invasion depth, and FIGO stage, the rise of radiomics—fueled by breakthroughs in AI and computational technology—now provides a more sophisticated way to predict these subtle pathological markers (13, 23–25). Prior work has used radiomics to forecast LVSI in cervical tumors (12–17, 26). Li et al. created an MR radiomics nomogram using 1.5T axial T1 CE-MRI scans from 105 patients to preoperatively predict LVSI, showing AUCs of 0.754 (training) and 0.727 (validation) (26). Wu et al. created and tested a multimodal MRI radiomics nomogram using 1.5T and 3.0T scans (T1WI, FS-T2WI, CE) from 168 cervical cancer patients across two centers to evaluate LVSI status. The model demonstrated strong predictive accuracy in both training and test groups (AUCs: 0.883 and 0.830) (13). Liu et al. created and validated a multiparametric MRI radiomics nomogram using sFOV HR-T2WI, DWI, and DCE-T1WI from 177 patients (3.0T MRI) to preoperatively predict LVSI, achieving AUCs of 0.838 (training) and 0.837 (testing) (16). Ma et al. developed and validated an MRI radiomics model using ADC, T2WI SPAIR, and T2WI sequences from 124 patients’ 1.5T MRI images to predict lymphovascular space invasion (LVSI) in resectable cervical cancer (CC). The nomogram achieved AUCs of 0.897 and 0.833 in the training and test cohorts, respectively (15). Xiao et al. created a radiomics nomogram using 1.5T multiparametric MRI from 233 stage IB–IIB cervical cancer patients (T1WI, FS-T2WI, DWI, ADC, CE) to predict lymph-vascular space invasion (LVSI). The model achieved AUCs of 0.78 and 0.82 in training and test cohorts, respectively (12). Huang et al. developed a multi-parametric MRI radiomics model using 3.0 T MRI, incorporating sFOV HR-T2WI, ADC, T2WI, FS-T2WI, and axial/sagittal T1c images to predict LVSI in 125 cervical cancer patients, achieving an AUC of 0.94 (17). Tumor heterogeneity significantly impacts treatment efficacy and prognosis. Wang et al. found that a radiomics model using tumor sub-regional habitats outperformed whole-tumor analysis in predicting LVSI among 300 cervical cancer patients across two institutions, achieving an AUC of 0.873 (14).

Despite radiomic research on multi-parametric MRI for Lymph-Vascular Space Invasion (LVSI) prediction in Cervical Cancer (CC), deep learning applications for LVSI prediction remain scarce. Shi et al. developed a radiomics model using both handcrafted and deep-learning features from 3.0T MRI T2WI scans of 160 patients. This intratumoral-peritumoral approach achieved AUCs of 0.859 (training) and 0.832 (testing) for improved predictive performance (27). Hua et al. applied a multiparametric MRI-based radiomics and deep learning approach to predict LVSI (21). The method, utilizing VGG-19, achieved AUCs of 0.842 in the training and 0.775 in the validation cohorts. It combined radiomics and deep features from tumor and peri-tumor tissues at varying radial distances (21). In another study, Jiang et al. employed a customized, end-to-end VGG19 network to distinguish lymph-vascular space invasion (LVSI) in 167 cervical cancer patients. Using both dynamic contrast-enhanced T1-weighted (DCE-T1) and T2-weighted (T2WI) MRI images, their model achieved an area under the curve (AUC) of 0.911, boasting a sensitivity of 0.881 and a specificity of 0.752 (19). Our predictive model, developed using multiparametric MRI sequences—including T2-weighted imaging (T2WI), apparent diffusion coefficient (ADC), and contrast-enhanced T1-weighted imaging (CE-T1WI)—demonstrated outstanding performance. Analyzing data from 155 patients scanned at both 1.5 T and 3.0 T, the model achieved an impressive AUC of 0.968 (95% CI: 0.938–0.999) in the training cohort and 0.859 (95% CI: 0.751–0.967) in the validation cohort. These results significantly outperformed prior studies, showcasing its superior predictive accuracy. The superior performance of our study is primarily attributed to two methodological innovations. First, the prediction model synergistically incorporates multi-parametric MRI sequences (including T2-weighted imaging, apparent diffusion coefficient mapping, and contrast-enhanced T1-weighted imaging), enabling holistic assessment of structural abnormalities, functional alterations, and tumor angiogenesis in relation to lymphovascular space invasion (LVSI). Secondly, the utilization of ResNet50 for deep feature extraction—contrasted with conventional VGG19—demonstrates enhanced capability in capturing LVSI-associated pathological patterns, likely attributable to its superior hierarchical feature representation and residual learning mechanisms.

Deep learning can autonomously extract features from medical images and construct models for classification tasks without human intervention. Yet this automated process presents a fundamental challenge–radiologists can’t easily decipher the logical reasoning behind the algorithm’s conclusions, creating what’s known as the “black box” dilemma in AI diagnostics (28, 29). This lack of interpretability is receiving increasing attention within clinical contexts, sparking growing demand for more transparent deep learning systems. While the “black box” of convolutional neural networks (CNNs) impedes traceability of decision rationales, Gradient-weighted Class Activation Mapping (Grad-CAM) (30) mitigates this limitation by generating visual saliency maps that localize prediction-critical regions. This visualization elucidates model attention mechanisms, aligning computational focus with clinically relevant image features. In medical imaging contexts, where precise lesion localization underpins diagnostic and therapeutic planning, Grad-CAM serves as a validation tool for highlighting pathognomonic abnormalities and morphometric alterations. In this study, we employed the Gradient-weighted Class Activation Mapping (Grad-CAM) algorithm to enhance model transparency and facilitate clinician understanding. In our ResNet50-based model, Grad-CAM mappings revealed modality-specific attention patterns: T2-weighted imaging (T2WI) heatmaps were predominantly localized to zones of cervical stromal disruption (indicating structural compromise); Apparent Diffusion Coefficient (ADC) mappings concentrated on functional impairment foci exhibiting diffusion restriction; Contrast-enhanced T1WI (CE-T1WI) activations highlighted regions with heightened tumor neovascularization at the lesion periphery. This tripartite attention alignment corresponds with the pathophysiological alterations implicated in lymphovascular space invasion (LVSI) of cervical carcinoma, where: Stromal ring rupture on T2WI may reflect tumor-induced matrix degradation; ADC restriction may signature correlate with intravascular tumor thrombus formation; Peritumoral neovascularization on CE-T1WI may indicate angiogenic switch activation.

In addition, this study explored the integration of the handcrafted radiomics and deep learning methods, and found that the fusion model demonstrated superior efficacy to single feature type models for the preoperative prediction of LVSI. This might be attributed to the restricted amount of information that can be offered by a single modality. The features extracted from diverse MRI sequences as well as different feature types might contain information that can reflect the tumor heterogeneity from various aspects. Moreover, by integrating the information from multiple modalities or even multiple feature types, the information on tumor heterogeneity can be mutually complementary, and finally improve the performance of the model. These findings suggest that radiomics and DL can be used as an important adjunct to significantly improve the accuracy of preoperative clinical decisions. The integration of deep learning with radiomics serves to advance the domain of personalized medicine (31). This integration of our model into clinical practice enables automated LVSI (lymphovascular space invasion) risk stratification following preoperative MRI examinations. These objective risk assessments, in conjunction with FIGO staging, can direct surgical decision-making—particularly by recommending fertility-preserving procedures for LVSI-low-risk patients—thereby minimizing overtreatment.

This research is not without its shortcomings. To begin with, the study was conducted as a retrospective single-center observational analysis, which raises concerns about potential selection bias in the data. The absence of multi-center collaboration limits the generalizability of the findings. Incorporating data from multiple institutions will be essential to verify the model’s accuracy and strengthen its validity; Additionally, although the deep learning features incorporated in this study show some predictive potential, the sample size is relatively small and the interpretability of the extracted deep learning features is limited, and subsequent studies need to incorporate more sample sizes to further improve the model fit and optimize the model efficacy.

In conclusion, the MRI-driven RDL model proves highly effective in predicting LVSI preoperatively for cervical cancer patients. Outperforming conventional methods, this model demonstrates significant clinical value through decision curve analysis, positioning itself as a promising non-invasive imaging biomarker. Its ability to enhance surgical planning could revolutionize treatment strategies while avoiding invasive procedures.

## Data Availability

The raw data supporting the conclusions of this article will be made available by the authors, without undue reservation.
